# Management of life-threatening anaphylaxis to enzyme replacement therapy in an infant with Pompe disease: a case report and literature review

**DOI:** 10.3389/falgy.2026.1757529

**Published:** 2026-03-16

**Authors:** Jueru Zhou, Qian Wang, Han Zhang, Yuqi Wang, Ziwei Wang, Fan Yang, Shiwei Yang

**Affiliations:** Department of Cardiology, Children’s Hospital of Nanjing Medical University, Nanjing, China

**Keywords:** anaphylaxis, CRIM-negative, enzyme replacement therapy, infusion-associated reaction, Pompe disease

## Abstract

**Background:**

Infantile-onset Pompe disease (IOPD) is a life-threatening lysosomal storage disorder, caused by deficiency of the acid alpha-glucosidase (GAA) enzyme. While enzyme replacement therapy (ERT) with recombinant human GAA (rhGAA) is the standard treatment, its efficacy in cross-reactive immunological material (CRIM)-negative patients is often complicated by severe infusion-associated reactions (IARs), posing significant challenges to management.

**Methods:**

We present a case of a CRIM-negative IOPD patient carrying a novel homozygous nonsense mutation in the GAA gene (c.2237G > A, p.Trp746*). Diagnosis was confirmed by significantly reduced GAA enzyme activity and genetic analysis. The patient was started on ERT using a desensitization protocol.

**Results:**

Despite initial desensitization, the patient experienced recurrent IARs, which progressed to life-threatening anaphylaxis (Grade 5, according to the WAO grading system for allergic reactions). Conventional preventive strategies including corticosteroid premedication, reduction of infusion rate, and dose reduction, failed to prevent the recurrences of severe reactions. A novel approach involving continuous epinephrine co-infusion with low-concentration rhGAA (1 mg/mL) was subsequently implemented, which may represent a potential rescue strategy to prevent further IARs and allow for continued ERT. Unfortunately, the patient ultimately died from severe pulmonary infection at 15 months of age.

**Conclusion:**

For CRIM-negative IOPD patients who develop refractory anaphylaxis to ERT, continuous epinephrine co-infusion represents a viable rescue strategy to facilitate treatment. This case underscores the critical need for early immune tolerance induction and the development of novel therapeutic modalities for this high-risk population.

## 1 Introduction

Pompe disease is a rare autosomal recessive lysosomal storage disorder caused by deficient activity of the acid alpha-glucosidase (GAA) enzyme resulting from pathogenic variants in the *GAA* gene (OMIM: 232300; EC 3.2.1.20). This enzymatic defect impairs lysosomal glycogen degradation, leading to glycogen accumulation primarily in skeletal muscle, cardiac muscle, and the liver, which in turn triggers a cascade of pathological events. The disease is classified into infantile-onset (IOPD) and late-onset (LOPD) based on age of onset, organ involvement, and progression rate. IOPD typically presents shortly after birth with hypotonia, feeding difficulties, hypertrophic cardiomyopathy, and macroglossia. Without intervention, patients usually succumb to cardiorespiratory failure within the first year of life.

Enzyme replacement therapy (ERT) with recombinant human GAA (rhGAA) is the cornerstone of treatment, supplementing the deficient endogenous enzyme (1). While the standard regimen is 20 mg/kg administered biweekly, escalated dosing (up to 40 mg/kg weekly) has shown improved outcomes in some studies. A major therapeutic challenge is the development of immune reactions, including infusion-associated reactions (IARs) and anti-rhGAA immunoglobulin G (IgG) antibodies, both compromising therapeutic efficacy and safety (2). Approximately 50% of IOPD patients experience IARs, which can affect multiple organ systems (cardiovascular, respiratory, dermatologic, and gastrointestinal) and may become life-threatening (2). Especially, CRIM-negative patients face a higher IAR risk due to poor ERT tolerance and robust antibody responses, whereas CRIM-positive patients have a lower risk with transient or no antibody production. Currently, there are no established guidelines for ERT discontinuation or rechallenge following severe IARs. Clinical management mainly relies on expert recommendations rather than standard guideline criteria (2, 3). This report presents a representative case to discuss management strategies for ERT-induced hypersensitivity reactions in IOPD.

## 2 Case report

### 2.1 Clinical presentation

The patient was born to non-consanguineous healthy Chinese parents. The maternal pregnancy was complicated by gestational diabetes mellitus and uterine fibroids, leading to premature delivery at 30⁺⁶ weeks due to premature rupture of membranes. At four months, the infant was hospitalized for cough and dyspnea and was concurrently diagnosed with cardiac hypertrophy. Despite nasal cannula oxygen support, the infant exhibited respiratory distress, inability to lift her head, and reduced limb movement. Physical examination revealed dull heart sounds, hepatomegaly (liver edge 3 cm below the right costal margin), bilateral lower extremity edema, and generalized hypotonia with decreased muscle strength in all extremities.

**Figure 1:**
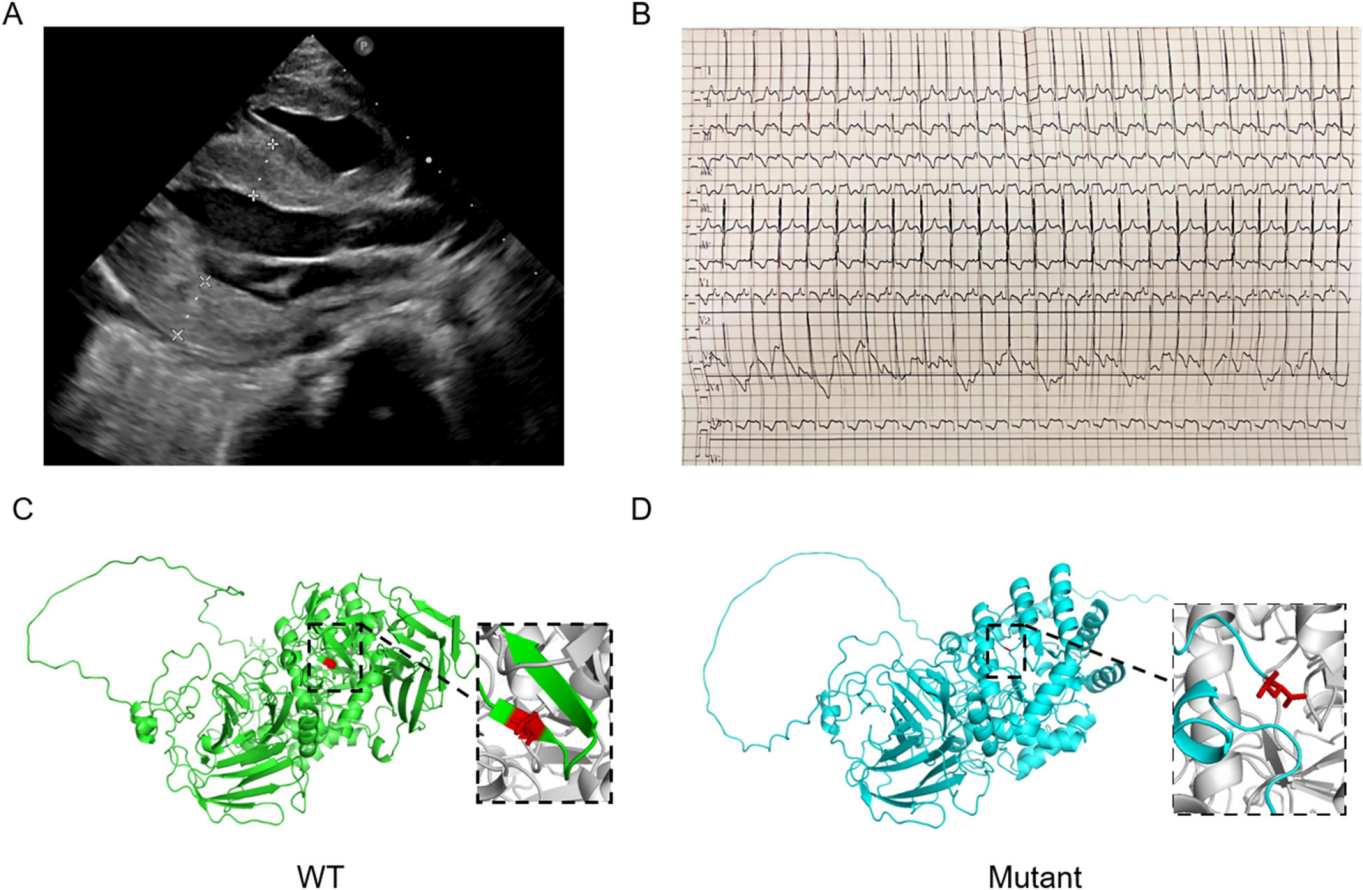
Clinical and genetic data of the patient. **(A)** The echocardiogram showed cardiac hypertrophy. **(B)** The Electrocardiogram showed left ventricular high voltage and diffuse ST-T segment changes. **(C,D)** The three-dimensional structures of wild- and mutant-type GAA protein.

Laboratory tests showed marked elevations in hepatic enzymes, creatine kinase, and brain natriuretic peptide (Table 1). Echocardiography demonstrated significant left ventricular and interventricular septal hypertrophy, increased myocardial echogenicity, reduced left ventricular ejection fraction (LVEF) (38.6%), and pulmonary hypertension (systolic pressure = 55 mmHg) (Figure 1A). Electrocardiogram indicated sinus rhythm, left ventricular high voltage, and diffuse ST-T segment abnormalities (Figure 1B). Based on these features, a genetic metabolic disorder, particularly Pompe disease, was suspected, and GAA enzyme activity was measured. Markedly decreased GAA enzyme activity (0.14 μmol/L/h; reference range: 1.29–20.34 μmol/L/h) and identification of a homozygous pathogenic GAA variant (c.2237G > A, p.Trp746*) confirmed the diagnosis of IOPD. Based on the identified homozygous nonsense GAA gene mutation, we inferred that our patient was CRIM-negative. The three-dimensional structures of wild- and mutant-type GAA were shown in Figures 1C,D.

**Table 1:** Changes in clinical parameters before and after ERT.

Parameters	Diagnosis	1 month	4 months	7 months	10 months
ALT (U/L)	105	156	176	258	283
AST (U/L)	216	297	287	542	781
CK (U/L)	1,404	427	613	1,544	1,839
BNP (pg/mL)	2,402	658	1,133	990.1	619.8
LVMI (g/m^2^)	275.9423	346.1961	256.7116	261.2749	244.8224
LVEF (%)	38.60	59.20	59.80	59.80	62.80

### 2.2 Treatment response and clinical course

Following diagnosis, the infant immediately began ERT with rhGAA at approximately 20 mg/kg, administered via a desensitization protocol as follows. First, based on the child's body weight of 5 kg, we dissolved 100 mg of the drug in 20.6 mL of sterile water for injection. Second, we diluted the dissolved drug with saline to a total volume of 50 mL, and it was administered via an anti-allergy infusion set. Third, the infusion rate was initiated at 6 mg/h, and gradually increased to 36 mg/h, with a total infusion duration of approximately 3.7 h. Due to severe concurrent infections and parental preference, the regimen was adjusted to weekly infusions. After one month of ERT, clinical improvements were observed, including reduced airway secretions, lower myocardial enzyme levels, and recovery of cardiac pump function (Table 1).

**Figure 2:**
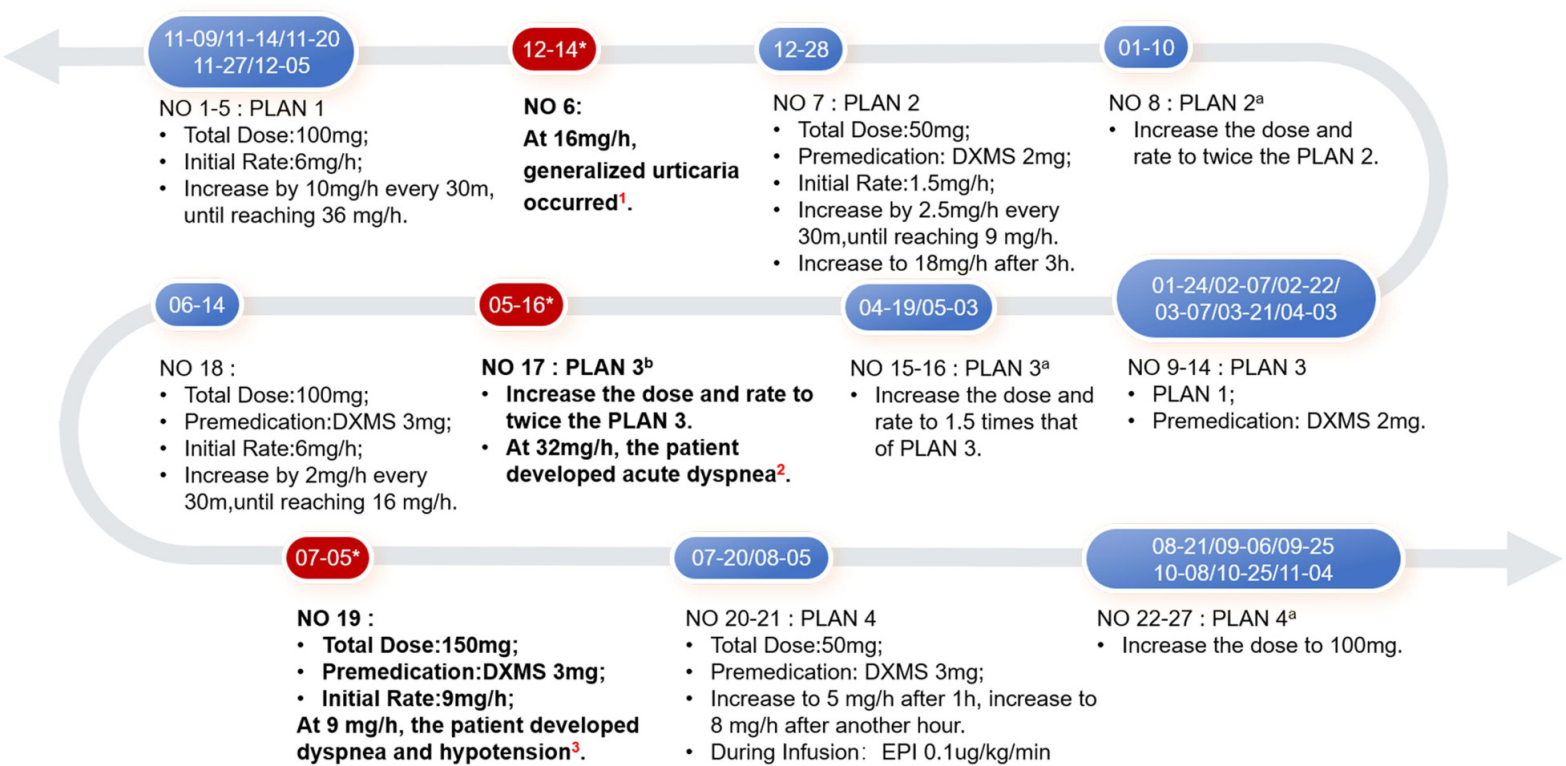
The ERT process of the patient and modified desensitization protocol. Key events ^1*^: Generalized urticaria occurred at 16 mg/h; urticaria recurred under DXMS 2 mg premedication and 10 mg/h infusion the next day. ^2*^: Acute dyspnea (SpO₂ 76-78%, RR 55 rpm, HR 140-160 bpm, BP 117/55 mmHg) developed at 32 mg/h; prompt interventions (oxygen therapy, intramuscular EPI, endotracheal intubation, and chloral hydrate enema) resolved symptoms. ^3*^: Dyspnea and hypotension (SpO₂ 80%, RR 65 rpm, HR 160-180 bpm, BP 65/45 mmHg) occurred at 9 mg/h; oxygen therapy and intramuscular EPI led to symptom resolution. (DXMS: dexamethasone; EPI: epinephrine).

However, the ERT course was complicated by multiple IARs (Figure 2). The first IAR occurred during the sixth ERT session (at approximately five months of age), manifesting as a generalized urticaria. The infusion was promptly discontinued, leading to symptom resolution. However, despite intravenous corticosteroid administration and rechallenge the next day, a maculopapular rash recurred. About one month later, following recurrent respiratory infections, ERT was resumed. To mitigate IAR risk, dexamethasone was administered as premedication, and the initial infusion rate was halved. After successful tolerance was established, the infusion rate was gradually escalated back to the original schedule, with dexamethasone continued as premedication over the subsequent 10 infusions.

By 10 months of age, the infant exhibited fewer respiratory infections and a significant improvement in myocardial hypertrophy (Table 1). However, during the 17th ERT session, the patient developed acute dyspnea and hypotension. Symptoms resolved following intramuscular epinephrine and resuscitative measures. During the 18th session (one month later), the total rhGAA dose was reduced to ∼10 mg/kg and the infusion rate was escalated more cautiously; this adjustment was successful. Nevertheless, three weeks later, the patient again experienced acute dyspnea and hypotension, requiring immediate intramuscular epinephrine rescue. Despite recurrent allergic reactions, ERT was considered essential due to persistent severe respiratory infections and rapid disease progression. A modified protocol was implemented: 10 mg/kg every other week, with dexamethasone premedication, gradual rate escalation, and concurrent low-dose intravenous epinephrine (0.1*μ*g/kg/min) during the infusion. No further IARs occurred under this regimen. Unfortunately, the patient eventually died of respiratory failure at 15 months of age.

## 3 Discussion

This case reports an IOPD infant harboring a novel GAA homozygous nonsense mutation (c.2237G > A, p.Trp746*). The clinical course was complicated by recurrent IARs, which were ultimately controlled using a protocol of continuous epinephrine co-infusion combined with a lower concentration of rhGAA. This case provides new clinical insights into the management of IARs in CRIM-negative IOPD patients.

ERT significantly improves the prognosis of IOPD. However, therapeutic efficacy depends on several factors, including age at initial treatment, severity of muscle damage, CRIM status, immunogenicity, and dosage (4). CRIM-negative infants are particularly susceptible to hypersensitivity reactions due to immune immaturity and the immunogenicity of exogenous GAA (5). An observational study from Italy reported allergic reactions in 8 of 28 IOPD patients (28.57%), including two cases (7.14%) of severe anaphylaxis (6).

Most CRIM-negative patients harbor homozygous or compound heterozygous GAA variants that preclude endogenous GAA production (7). Consistent with this, our patient carried a homozygous nonsense GAA mutation and her poor outcome was likely compounded by the CRIM-negative phenotype. Although a desensitization protocol with gradual escalation of drug dosage was initiated from the outset, a Grade 1 IAR (generalized urticaria) occurred during the sixth infusion. A modified regimen involving corticosteroid premedication and a reduced initial infusion rate was initially successful for several months. However, the patient subsequently developed recurrent Grade 5 (acute dyspnea) reactions. Despite intensification of glucocorticoids, reduction of the infusion rate, and dose reduction, life-threatening anaphylaxis recurred. As epinephrine is first-line therapy for severe allergic reactions (8), we introduced a novel strategy of concurrent intravenous epinephrine during ERT, which successfully prevented further IARs. Though epinephrine is typically administered via intramuscular injection for acute reactions, considering the patient's young age, low body weight, and severe allergic reaction, repeated intramuscular injection may be technically challenging and increase the child's distress, also compromising therapeutic efficacy due to poor absorption at the injection site. In contrast, under monitoring, continuous intravenous infusion was delivered through an established venous access, ensuring treatment continuity with fewer procedure-related risks. Our case highlights the feasibility of continuous epinephrine infusion as an adjunctive approach during ERT for severe IARs, as evidenced by the observed clinical efficacy and safety in our patient. Notably, the severe reaction emerged when the drug concentration reached 4 mg/mL (within the recommended range (9), whereas maintaining a concentration of 1 mg/mL prevented IARs recurrence. This suggests that lower concentrations may reduce the risk of hypersensitivity when fluid tolerance allows. However, based on our single-case report, we cannot definitively conclude whether hypersensitivity was primarily driven by drug concentration, total dose, or infusion rate. We acknowledge this uncertainty and emphasize that this finding requires validation in larger cohorts or studies to clarify the relative contributions of these factors to IARs.

Hypersensitivity reactions to ERT may be IgE- or non-IgE-mediated. Beyond premedication, infusion rate adjustment, and desensitization, tranexamic acid (500 mg/day) has been proposed for vascular edema or complement-mediated IARs, as it inhibits plasmin-mediated kallikrein activation and interrupts the kinin generation cascade (10). Anti-IgE monoclonal antibodies (e.g., omalizumab) reduce allergic reactions by binding free IgE and preventing its engagement with Fc*ε*RI on mast cells and basophils (9). In one reported IOPD case, omalizumab enabled sustained ERT, though long-term use appears necessary to maintain tolerance (11). In our case, anti-rhGAA IgE testing was not performed, and omalizumab and tranexamic acid were not used, because of their limited approved indications and the patient's relatively young age, without safety and dosage data specific to children. Though both immune tolerance induction (ITI) and desensitization therapy aimed to protect PD patients from IARs, they are distinguished. ITI represents a prophylactic strategy for preventing IARs, especially in CRIM-negative IOPD patients, which is administered before ERT initiation. Differently, desensitization therapy is an intervention for patients who have experienced IARs to prevent the recurrence of IARs. Early initiation of ITI has been established as a standard treatment protocol in many specialized PD centers for high-risk patients (12–14). Alternatively, a proactive ITI regimen (e.g., rituximab, methotrexate, intravenous immunoglobulin) has been shown to promote B-cell reconstitution and improve outcomes in IOPD, whereas T cell-targeted immunosuppressants (e.g., mycophenolate mofetil and cyclosporine A) have failed to induce durable tolerance to rhGAA (15–17). Given the acute severity of the patient's reaction, we may consider prior continuous epinephrine infusion over these ITI regimens, which typically require longer efficacy periods. Therefore, in high-risk CRIM-negative patients, preemptive implementation of standard ITI protocols (when feasible) combined with epinephrine co-infusion and B-cell–targeted immunomodulation may help avert severe IARs.

Recent advances have expanded the Pompe therapeutic landscape to include gene therapy, molecular chaperones, antisense oligonucleotides, and substrate reduction therapy (18–20). Gene therapy can deliver the functional GAA gene, restore enzyme activity, and potentially mitigate immunogenicity. For our patient, early intervention with gene therapy might have prevented ERT-associated IARs and more effectively attenuated disease progression.

This study has several limitations. First, this study is a single-case report and lacks statistical power. Furthermore, the patient's CRIM-negative status was predicted solely from homozygous nonsense GAA mutations rather than confirmed by experimental assays. Additionally, the absence of rhGAA IgE/ IgG antibody measurements, as well as the untested acute-phase serum tryptase levels, has severely impeded in-depth elucidation of IARs pathogenesis and the acquisition of direct evidence for mast cell activation. Last, due to the single-case design, this study cannot definitively distinguish whether IARs are driven primarily by drug concentration, total dose, or infusion rate; the observed correlation between low rhGAA concentration and reduced hypersensitivity risk provides a preliminary indication and needs to be validated in larger clinical cohorts.

## 4 Conclusion

In conclusion, our report delineates the challenging clinical course of a CRIM-negative IOPD patient who developed recurrent, severe anaphylaxis to ERT. As a single-case report, our experience suggests that incorporating continuous epinephrine infusion and lower rhGAA concentration can successfully mitigate IARs and permit sustained ERT, offering a practical strategy for managing similar refractory cases, whereas this approach requires further validation in larger cohorts or controlled studies. Besides, we emphasize the importance of early risk stratification via CRIM status, consideration of preemptive immunomodulation, and the urgent need for emerging therapeutics like gene therapy, which hold promise for averting hypersensitivity reactions and improving long-term outcomes in high-risk CRIM-negative IOPD patients.

## Data Availability

The original contributions presented in the study are included in the article, further inquiries can be directed to the corresponding authors.
